# Comparing Mobile Health Strategies to Improve Medication Adherence for Veterans With Coronary Heart Disease (Mobile4Meds): Protocol for a Mixed-Methods Study

**DOI:** 10.2196/resprot.7327

**Published:** 2017-07-18

**Authors:** Linda G Park, Eileen G Collins, Janet K Shim, Mary A Whooley

**Affiliations:** ^1^ Department of Veterans Affairs Community Health Systems University of California, San Francisco San Francisco, CA United States; ^2^ Edward Hines Jr., VA Hospital Department of Biobehavioral Health Science University of Illinois at Chicago Chicago, IL United States; ^3^ University of California, San Francisco Social and Behavioral Sciences San Francisco, CA United States; ^4^ Department of Veterans Affairs Department of Medicine, Epidemiology and Biostatistics University of California, San Francisco San Francisco, CA United States

**Keywords:** text messaging, mobile application, mobile health, cardiovascular disease, medication, adherence

## Abstract

**Background:**

Adherence to antiplatelet medications is critical to prevent life threatening complications (ie, stent thrombosis) after percutaneous coronary interventions (PCIs), yet rates of nonadherence range from 21-57% by 12 months. Mobile interventions delivered via text messaging or mobile apps represent a practical and inexpensive strategy to promote behavior change and enhance medication adherence.

**Objective:**

The Mobile4Meds study seeks to determine whether text messaging or a mobile app, compared with an educational website control provided to all Veterans, can improve adherence to antiplatelet therapy among patients following acute coronary syndrome (ACS) or PCI. The three aims of the study are to: (1) determine preferences for content and frequency of text messaging to promote medication adherence through focus groups; (2) identify the most patient-centered app that promotes adherence, through a content analysis of all commercially available apps for medication adherence and focus groups centered on usability; and (3) compare adherence to antiplatelet medications in Veterans after ACS/PCI via a randomized clinical trial (RCT).

**Methods:**

We will utilize a mixed-methods design that uses focus groups to achieve the first and second aims (N=32). Patients will be followed for 12 months after being randomly assigned to one of three arms: (1) customized text messaging, (2) mobile app, or (3) website-control groups (N=225). Medication adherence will be measured with electronic monitoring devices, pharmacy records, and self-reports.

**Results:**

Enrollment for the focus groups is currently in progress. We expect to enroll patients for the RCT in the beginning of 2018.

**Conclusions:**

Determining the efficacy of mobile technology using a Veteran-designed protocol to promote medication adherence will have a significant impact on Veteran health and public health, particularly for individuals with chronic diseases that require strict medication adherence.

**Trial Registration:**

ClinicalTrials.gov NCT03022669

## Introduction

### Coronary Heart Disease and Medication Adherence

Coronary heart disease is the leading cause of death in Veterans and affects 15.5 million people in the United States [1,2]. Although coronary heart disease claims the lives of one out of every seven Americans [2], the decline in acute coronary syndrome (ACS) and coronary heart disease-related deaths over the past four decades has been attributed to the availability of critical pharmacologic and invasive therapies (ie, percutaneous coronary intervention [PCI]) [3-6]. After PCI with drug-eluting stents, premature discontinuation of antiplatelet (thienopyridine) medications is strongly associated with in-stent thrombosis, leading to myocardial infarction (MI) and death [7,8]. Despite the critical nature of taking antiplatelet medications to prevent life-threatening complications, one in seven MI patients treated with drug-eluting stents no longer take antiplatelet medications at 30 days [8].

Medication nonadherence is a pervasive public health problem among Veterans and non-Veterans [9-12]; however, Veterans suffer from increased morbidity and mortality from chronic diseases compared to non-Veterans [13]. Medication nonadherence is the number one problem in treating illnesses, as more than half of individuals with chronic diseases do not take any or all of their medications correctly [14,15]. Medication nonadherence in patients with coronary heart disease is closely linked to adverse clinical outcomes such as rehospitalization and mortality [16-18]; therefore, effective strategies are needed to improve adherence behaviors. Moreover, medication nonadherence results in an estimated US $290 billion in health care costs in the United States and is a tremendous burden on the health care system [19]. Mobile technology may represent an effective strategy to improve medication adherence during the critical time period of one year post-ACS/PCI.

### Mobile Health

In the past decade, mobile health (mHealth) has been introduced as a mechanism to enhance medication adherence and demonstrates strong potential to promote behavior change. The use of technology can facilitate adoption and integration of medication adherence by promoting behavioral strategies via health messaging, emphasizing healthy habits, tracking goals, and giving incentives for behavior change [20]. While other behavioral and educational strategies have had disappointing results in adherence and treatment outcomes over many decades [21,22], the use of technology may provide an innovative, practical, personalized, and inexpensive approach to promote medication adherence. Diverse solutions are required with tailored theory-based interventions for different populations and conditions because the reasons for medication nonadherence are complex and multifactorial [23,24]. Experts agree that a patient-centered approach is critical to increase patients’ knowledge about medication management [25].

Both text messaging and mobile apps are two popular forms of mHealth that provide convenient, inexpensive, and nonintrusive engagement with individuals. The use of mobile technology can particularly benefit those who have significant barriers to taking medications, such as confusion about which medications to take, forgetfulness, and lack of social support. Text messaging is more widely used by all age groups; however, mobile apps offer many more features than text messaging, and can harness the full sensing and computational capacity of mobile devices to collect and analyze health-related data in real time to deliver health and behavioral interventions [20,26].

Text messaging is popular, fast, direct, efficient, user-friendly, traceable, and provides easy data transfer [27]. Text messaging is also nonintrusive, relatively simple, and lower in cost compared to mobile voice communication [28]. In a systematic review of medication adherence studies using text messaging, we found that most interventions (18/29) resulted in improved medication adherence [29]. This review did not examine any studies related to coronary heart disease, but gaps in the literature were discussed, such as understanding factors that are needed to maintain engagement (eg, content and frequency of text messaging). In contrast to text messaging, apps can offer interactivity, gaming, and feedback. Apps have the advantages of delivering interventions to an unlimited number of individuals in a cost-effective manner with favorable cost utility [30]. While Internet interventions can be delivered via traditional desktop and laptop computers, users have the capacity to interact with mobile interventions more frequently and in the context of the behavior of interest [31]. The trend of desktop use for research/program interventions is sharply declining in our current generation, given that most people carry and access their mobile phones throughout the day for various reasons. Using real-time sensing technologies available on mobile phones, health behavioral change interventions can be delivered based on time/location, psychological state, physiological state, social context, activity level, and patterns [32].

### Preliminary Studies

In a pilot randomized controlled trial (RCT) from 2012-2013, we compared antiplatelet and statin adherence among patients with coronary heart disease in a 30-day study who received: (1) text messaging for medication reminders and education, (2) educational text messaging only, or (3) no text messaging [33]. Among 90 patients (76% male, mean age 59.2 years), electronic devices revealed that patients who received text messaging for antiplatelets had a higher percentage of correct doses taken (*P*=0.02), percentage number of doses taken (*P*=0.01), and percentage of prescribed doses taken on schedule (*P*=0.01) [33]. Text messaging response rates were higher for antiplatelets than statins (*P*=0.005), and self-reported adherence revealed no significant differences among groups [33]. We concluded that the use of text messaging for the medication reminders/education and education text messaging only groups (compared to no text messaging for the control group) increased adherence to antiplatelet therapy by 15% to 17%, respectively. We established feasibility and high satisfaction and concluded that mHealth interventions show promise in promoting medication adherence.

### Aims

**Figure 1:**
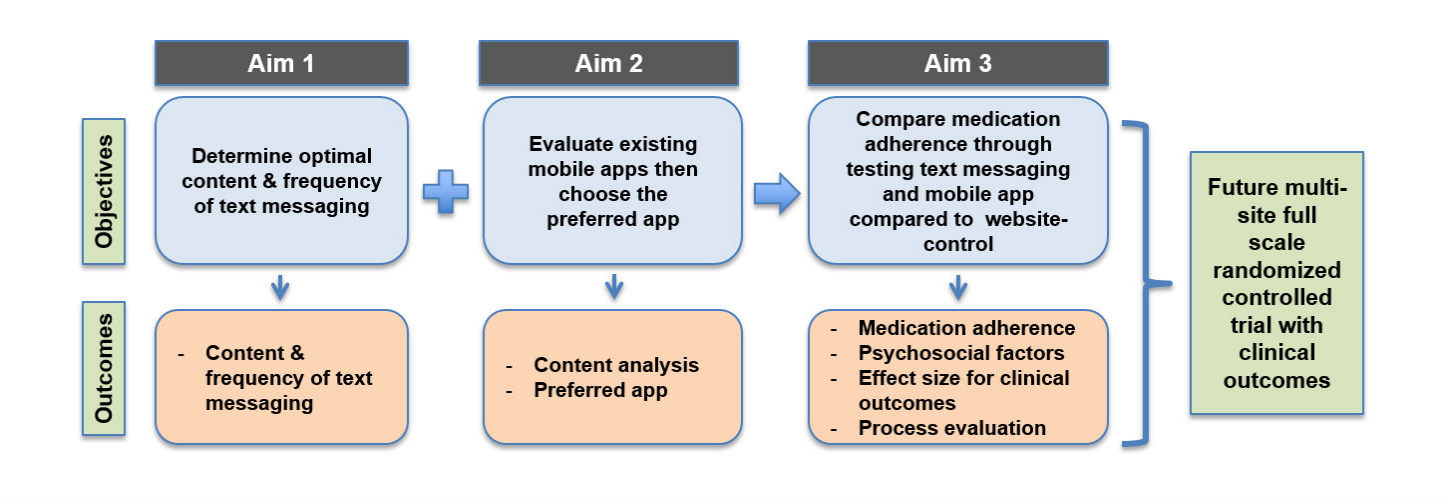
Aims, objectives, and outcomes.

The proposed research study, Mobile4Meds, seeks to determine whether text messaging or a mobile app (compared with an educational website control provided to all Veterans) can improve adherence to antiplatelet therapy among patients post-ACS/PCI. There are three topics and aims of this proposed study. First, using *Text Messaging* we aim to determine preferences for content and frequency of text messaging to promote medication adherence. Second, using *Mobile Apps* we aim to identify the most patient-centered (user-friendly, engaging, personalized) mobile app that promotes medication adherence with stratification by low/high mobile phone use and sex. Finally, we aim to examine *Medication Adherence* by comparing adherence to antiplatelet medications upon hospital discharge from ACS/PCI via: (1) text messaging, (2) mobile app, or (3) website-control. Figure 1 illustrates the aims, objectives, and outcomes of the proposed research study. Our long-term goal is to conduct a multi-site full scale RCT to determine clinical outcomes of emergency department utilization, rehospitalization, and cardiovascular (and all-cause) morbidity and mortality.

## Methods

### Theoretical Framework/Behavioral Change Model

**Figure 2:**
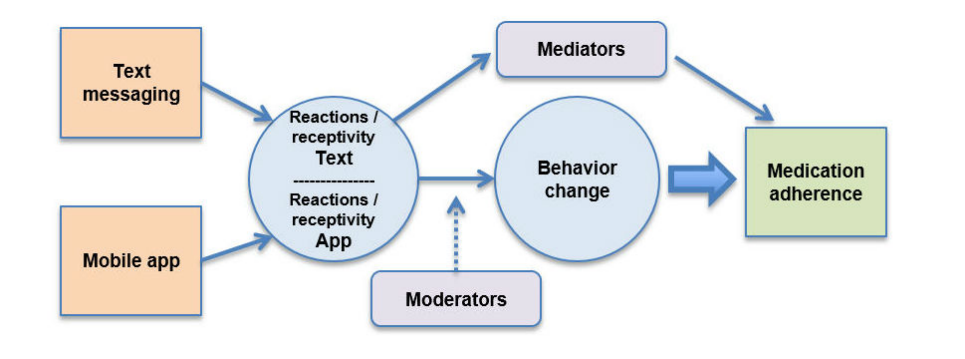
Conceptual model of behavior change for medication adherence.

To date, most mHealth studies have not been based on behavioral health theories [29,31]. In a systematic review of text messaging studies on medication adherence in various acute and chronic conditions, we found that only 5 of 29 studies used theory to guide research [29]. Numerous precipitating factors and barriers affect medication adherence, and this complex process can be better understood when examining theoretical relationships and process. The conceptual model that explains the relationship between variables in this study is depicted in Figure 2. No single theory can completely explain the processes that lead to medication adherence, so we will use a theory-informed approach to complete the aims for this study. We will explore the general components of theory that are closely linked with behavioral change interventions (eg, perceived norms/threats, health beliefs, self-efficacy, cues to actions).

**Figure 3:**
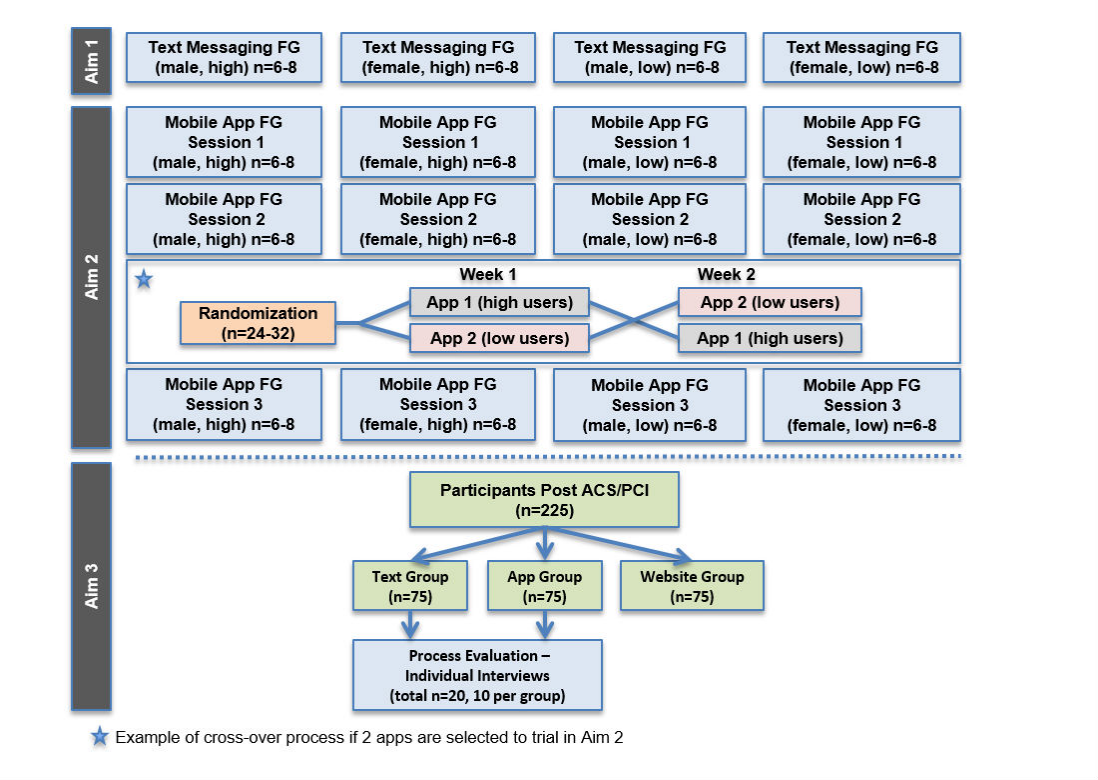
Overview of study structure and sample. FG: focus group.

### Research Design

Mobile4Meds is a mixed-methods study using both qualitative and quantitative research methods. Engaging patients initially through qualitative methods will provide opportunities for: (1) better translation, dissemination, and use of research results; (2) better evidence to inform guidelines; (3) targeted quality improvement; and (4) using research to address concerns of diverse patients [34]. Figure 3 provides an overview of the study structure and participants involved in each of the aims.

### Aim 1: Text Messaging Focus Groups

#### Design, Sample, Setting

We will use focus groups to fulfill the objectives of Aim 1. Each focus group will have 6-8 participants, which is a size that allows for easy exchanges of ideas and a diversity of perspectives to be represented. We will recruit a convenience sample of 24-32 participants for four focus groups. Aims 1 and 2 will include the same participants who will attend a total of four focus group sessions (Figure 3).

The participants from San Francisco Veterans Affairs Medical Center (SFVAMC) are likely to be predominantly male; therefore, we will likely recruit one group of female Veterans from SFVAMC and one focus group of female non-Veterans from John Muir Medical Center (JMMC) in the East Bay. The four groups will account for variability in mobile phone use (low/high) and sex. Inclusion criteria for Aims 1 and 2 include: (1) >21 years of age, (2) history of ACS or PCI within one year, and (3) current/former antiplatelet (thienopyridine) prescription. Exclusion criteria are: (1) cognitive impairment, and (2) lack of English proficiency/literacy.

#### Methods

##### Preliminary Development of Text Messaging

We will explore different content for the development of a text messaging intervention to determine the ideal tailored messages for delivery in the RCT (Aim 3). After studying various behavioral change theories and frameworks, we will create a text messaging intervention with the underpinnings of common behavioral change theories. A library of 80-100 sample text messages that apply personalized content will be created to enhance medication adherence. We found that education alone increased medication adherence in prior studies, so we will deliver cardiovascular risk reduction messages along with messages regarding medications. Topics will include medications, diet, exercise, risk factors for coronary heart disease (eg, smoking, obesity), depression, stress management, and preventive care. Participants will choose the frequency and timing of text messaging. An example of an automated medication text messaging may be, “John, take your Plavix 75mg at 9am. Confirm with 1.”

##### Recruitment

We will distribute written materials such as flyers and brochures to advertise voluntary participation in the research study. The research team (principal investigator and research assistant [RA]) will consult with the cardiac rehabilitation staff at SFVAMC and JMMC to identify individuals who are willing to participate in a research study. The research team will meet with interested individuals and provide verbal and written explanation of the study. Participants will sign a written informed consent document.

##### Group Assignment

Upon recruitment into the study, participants will complete a sociodemographic questionnaire and a brief survey that will determine if he/she is a *low* or *high* mobile phone user. Separating participants into groups will help facilitate identification of common ideas and goals that low/high mobile phone users and men/women may address during the focus groups. We will use maximum variation sampling to ensure variability in age, gender, ethnicity, and low/high mobile phone use.

##### Focus Groups

In Aim 1, the focus group sessions will address ideal content and frequency of text messaging. First, we will give an introduction of the study aims and purpose of the focus group. Second, we will discuss the needs, preferences, perceived facilitators, and perceived barriers of using text messaging for medication adherence. Third, we will review sample text messaging, and we will ask participants which content and frequency of text messaging are the most effective and least intrusive. Finally, all participants will be able to suggest text messaging to create the most personalized messages.

##### Data Collection

All focus groups will be audiotaped, and the RA will take field notes on individual and group reactions that are not captured by the recording. We will complete field notes at the conclusion of each session, and all sessions will be transcribed verbatim.

#### Analysis

Thematic analysis will include reading and coding the completed field notes and interview transcripts. We will create an initial set of codes that account for the study aims and other topics that emerge from the data. Some codes will be generated deductively, while others will be generated inductively based on themes and issues that emerge from ongoing coding. We will compare coding, resolve differences, and revise the coding scheme. We will assess intercoder reliability by having 10% of the data cross-coded. The key outcomes will be content and frequency of text messaging, particularly focusing on how preferences vary across groups. We will use ATLAS.ti (Thousand Oaks, CA) for all qualitative analyses to determine the preferred app for Aim 3.

### Aim 2: Mobile Apps: Focus Groups

#### Design, Sample, and Setting

Aim 2 consists of Aim 2a (*content analysis*) and Aim 2b (*usability testing*). The usability study will include the same individuals from the focus groups in Aim 1 who are stratified by low/high mobile phone use and sex, to accommodate easy exchanges of ideas and a diversity of perspectives. In contrast to Aim 1, there will be three focus group sessions to fulfill Aim 2. Recruitment, group assignment, and data collection will be the same for Aim 2a and Aim 2b.

##### Aim 2a – Content Analysis

###### Methods

We will conduct a content analysis approximately three months prior to the focus groups. The first phase will involve a comparative, descriptive assessment of smartphone apps in the categories of health and medications. To ensure a comprehensive search, the research team will search for medication adherence-oriented apps, provider websites, and app sources that are available in Apple iTunes, Android Marketplace, and Blackberry App World Store.

We will provide a comparative, descriptive assessment of the top 10 smartphone apps related to medication adherence. The content analysis will examine whether each app applies constructs from behavioral change theory. We will assess each of the selected apps using a validated behavioral theory content survey by Doshi et al [35]. The survey assesses the presence of essential constructs of four major theories of behavior change: Health Belief Model, Transtheoretical Model, Theory of Reasoned Action/Planned Behavior, and Social Cognitive/Social Learning Theory [35]. After content analysis is completed, we will rank the top 10 medication adherence apps and record usability, application of theory, user reviews, star ratings, and the total number of downloads.

###### Analyses

We will analyze the content analysis by following the scoring instructions of the Behavioral Theory Content Survey for the top 10 mobile apps [35]. The Behavioral Theory Content Survey will include 20 items from the following domains listed in Table 1. Mean survey score and standard deviation (SD) will be computed across the top 10 apps based on a total of 20 items from the 5 domains, each accounting for 1 point (yes=1, no=2) [35]. We will then provide a descriptive evaluation of the apps based primarily on survey scores (mean, SD) and subsequent rankings [36]. Interacting with the mobile app will include a dynamic process of iterative adjustments in response to user input, key features, and functionality that may not be apparent upon initial use and subsequent input [36].

**Table 1:** Behavioral strategies included in the Behavioral Theory Content Survey [35,36].

Domain	Example
1. Knowledge	General information
2. Cognitive strategies	Perceived benefits/barriers/risks, self-efficacy
3. Behavioral strategies	Self-monitoring, time management, social support
4. Emotion-focused strategies	Stress management
5. Therapeutic interventions	Motivational readiness

##### Aim 2b - Usability Testing: Methods

###### Mobile App Focus Groups – Session 1 (Formative Stage)

After the research team identifies the top 10 medication reminder apps through content analyses, the focus group for Session 1 will help to determine the top 2-4 apps the participants will use for one week at a time. Four different groups will be stratified by low/high mobile phone use and sex (Figure 3). We will briefly demonstrate the apps for participants on a large screen and ask participants to engage with the preloaded apps on our research smartphones. We will assess participants’ impressions of the apps and ask them to score the apps (range 1-5) so we can determine the ideal number of apps to trial from the distribution of rankings. Participants’ comments and overall scores will help determine the preferred apps.

###### Session 2 (Exploratory Stage)

During the focus group for Session 2, the majority of time will be spent on group assessment of overall experience with mobile apps in general. After the group assessment, we will include introduction of the preselected apps from Session 1. The selected apps from Session 1 will be downloaded to the patients’ mobile phones devices. Patients will spend approximately 10-15 minutes experimenting with and evaluating the selected apps. We will conduct observations of patients as they interact with the mobile apps. Participants will be randomly assigned to use the first app in Session 2 and use that app for one week, then switch to the other app(s) in subsequent weeks. Figure 3 displays a scenario in which participants will be randomly assigned to use two mobile apps for two weeks; however, participants may trial between 2-4 mobile apps for one week each, depending on the results of the Mobile App Session 1 focus groups.

Participants will record impressions in a diary while using the apps. We will contact the participants by text/email/ phone-call/visit within two days after using each mobile app to troubleshoot any difficulties. At the start of each week, we will contact participants to remind them to switch over to the next mobile app.

###### Session 3 (Usability Stage)

After the trial period with mobile apps, the focus group for Session 3 (which assesses usability) will include: (1) follow-up on patients’ experience and preferences with using the selected mobile apps, and (2) completion of a 10-item System Usability Scale to provide a quantitative evaluation of the mobile apps to supplement the focus groups and interviews [37]. A winning app will be selected for the RCT.

##### Usability Testing: Analysis

The three focus group sessions for Aim 2 will be transcribed, coded, and analyzed in the same manner as in Aim 1. The System Usability Scale used in the Mobile App Session 3 focus group will yield a single number from 0 to 100 representing a composite measure of the overall usability of mobile apps being tested [37]. The scale will be scored as directed by the original authors.

### Aim 3: Medication Adherence Randomized Controlled Trial

#### Design

The research design for Aim 3 is an RCT that lasts 12 months. In contrast to Aims 1 and 2 that included participants who were post-ACS/PCI within one year and recruited from a cardiac rehabilitation program, participants for Aim 3 will be recruited from the hospital after ACS/PCI.

#### Sample Size Estimation

We plan to enroll 225 total participants (75 participants per group). Allowing for a 15% attrition rate over the 12-month study, we conservatively estimate that this recruitment target will result in a total sample size of n=192, which will give 86% power to detect a medium effect size (half-SD difference) between at least one treatment group and the control group using Dunnett’s method to adjust for multiple comparisons. This sample size would give more than 95% power to detect an effect size of d=0.69, as found in our previous clinical trial [33]. Analysis of the longitudinal data points with mixed-effects models will confer additional power.

#### Sample

The convenience sample will consist of 159 male and female Veterans from SFVAMC and 66 female participants (29% of sample) from JMMC. A sample size of 225 is considered to be feasible considering the number of post-ACS/PCI patients who are treated at both institutions. We have incorporated a 29% oversample of women to ensure a sufficient sample size to examine subgroup differences in intervention effects, given the low enrollment of female Veterans in Veterans Health Administration (6%) [38]. Inclusion criteria include: (1) >21 years of age, (2) recent ACS or PCI within one week, and (3) new antiplatelet (thienopyridine) prescription. Exclusion criteria are: (1) cognitive impairment, and (2) lack of English proficiency/literacy. For the process evaluation, we will recruit a subset of 10 participants from each experimental group (text messaging and mobile app; total n=20) for in-depth individual interviews.

#### Settings and Recruitment

We will recruit from SFVAMC and JMMC inpatient units using two recruitment strategies. First, we will inform the cardiologists and nurses in the cardiac units at the hospital sites about the study so they can identify patients who meet eligibility criteria. A patient-friendly handout will be made available for the health care team to give to interested participants. Second, we will obtain a daily list of patients who had PCI and their electronic medical records will be screened for eligibility in the study using a checklist to examine the inclusion and exclusion criteria. Participants who agree to participate will provide written informed consent.

#### Randomization

Group assignment will be generated by random allocation sequence using random blocks stratified within recruitment site (SFVAMC or JMMC) of either three or six, which will be prepared by the study biostatistician. The research team will assign patients to their groups by distributing presealed envelopes in consecutive, numbered order. Due to the nature of the study design, the research team and participants cannot be blinded to the intervention once group assignment is determined.

#### Baseline Data Collection

After written consent is obtained, we will collect clinical data by reviewing electronic medical records. Participants will complete the following baseline questionnaires: (1) sociodemographic information and medical history, (2) mobile phone use, and (3) Mini-Cog Test [39]. All instruments and frequency of administration are described in Table 2.

**Table 2:** Measurement of medication adherence with intervals.

Measure	Description	Rationale	Months					
			0	1	3	6	9	12
Key Primary Endpoint								
Medication Event Monitoring System (MEMS) [40]	Electronic pill caps time stamp when the bottle is opened, correlating with intake of medication. Primary outcome is percentage (0-100%) of prescribed doses taken	Most objective, noninvasive method and considered the “gold standard”		x		x		x
Secondary Patient-Reported Endpoints								
MEMS [40]	Percentages (0-100%) of (1) days correct doses are taken, and (2) doses taken on schedule	Most objective, noninvasive method and considered the “gold standard”		x		x		x
Morisky Scale [41,42]	Eight-item Morisky Medication Adherence Scale (MMAS-8) self-reported questionnaire on adherence behavior for a range of scores 0-8, with higher scores indicating poor adherence (Cronbach alpha=0.83).	Most popular scale and well-validated instrument with high reliability	x	x		x		x
7-Day Recall	One item asking how many days in the past week patient remembered to take medications. Final outcome is a percentage (0-100%).	Most commonly used method in the clinical setting	x	x		x		x
Secondary Objective Endpoints								
Veterans Affairs Corporate Data Warehouse (Veterans)	Veterans Affairs software program that provides detailed information on inpatient and outpatient medications. Final outcome is a percentage (0-100%) of days covered during 90-day period.	Assesses adherence via 90-day refill patterns (does not require active participation)		x	x	x	x	x
Peoplechart Meds Incontext (non-Veterans)	Private software program that uses refill data directly from retail pharmacies and Personal Benefits Manager. Final outcome is a percentage (0-100%) of days covered during 90-day period.	Assesses adherence via monthly refill patterns (does not require active participation)		x	x	x	x	x

#### Intervention

We will conduct an RCT with random assignment to one of three groups: (1) text messaging, (2) mobile app, or (3) website-control, for a total of 12 months. Both intervention groups will also be offered the patient education website that the control group will be given. The *text messaging group* will receive text messages with content and frequency informed by Aim 1. We will use the Veterans Affairs (VA) *Annie* text messaging program. For non-Veteran females, we will use a text messaging program from CareSpeak Communications. The *app group* will use the preferred app that is selected in Aim 2. The major purpose of the app is to remind participants to take medications; however, the app will likely include other features such as providing educational messages. The *website group* will be offered the American Heart Association patient education website (My Life Check - 7 Steps To Healthy Living) and will serve as an active control group [43]. The website will be offered to participants in all three groups. The *7 small steps to big changes* are to manage blood pressure, control cholesterol, reduce blood sugar, get active, eat better, lose weight, and stop smoking.

#### Follow-Up Visits

Subsequent in-person follow-up visits at 1, 6, and 12 months will take place in participants’ homes or other mutual meeting places as requested. Follow-up visits will be scheduled at times that are convenient for participants to meet.

### Primary Outcome/Other Variables and Instruments

#### Primary Outcome: Medication Adherence

Medication adherence will be assessed with five different measures at various intervals to validate patterns of adherence, including: Medication Event Monitoring System (MEMS; key primary endpoint being percentage of prescribed doses taken), Morisky Medication Adherence Scale (MMAS-8), 7-day recall, VA Corporate Data Warehouse (Veterans), and Peoplechart Meds Incontext (non-Veterans; Table 2). In-person follow-up visits will occur at 1, 6, and 12 months, while monitoring of adherence through refill data from computer software will be done monthly to reduce participant burden.

MEMS technology uses integrated microcircuits with bottle caps that record the date and time that an individual opens a pill bottle. Participants will be given one bottle to store their antiplatelet medication, and will be directed to store their other medications as they normally would. For those who use 7-day pill boxes, we will emphasize the need to use the MEMS bottle for the antiplatelet medication. Data will be wirelessly transferred to a MEMSCap reader, which is a small device that connects to a computer software program.

Refill data from computer software will also allow us to monitor prescription adherence passively, without requiring patient participation. We will use administrative data from the national VA repository (VA Corporate Data Warehouse) to provide adherence to antiplatelet medication for Veterans. For non-Veteran females from JMMC, we will use an equivalent data source through Peoplechart Meds Incontext, which is a software program that tracks adherence through sanctioned prescription data sourced directly from retail pharmacies and Prescription Benefits Managers (ie, mail-order companies).

#### Other Variables and Instruments

We will examine other variables (health-related quality of life, social support, self-efficacy, depression, anxiety, and post-traumatic stress disorder) and their relationship to medication adherence. Patients will complete the instruments at baseline, 1, 6, and 12 months (Table 3).

**Table 3:** Instruments used to assess additional variables.

Measure	Description	Months					
		0	1	3	6	9	12
Health-related quality of life	The 12-Item Short Form Health Survey (SF-12) is the shorter version of SF-36, which measures functional health and well-being (relative validity median 0.67 and 0.97 for the physical and mental components, respectively) [44].	x	x		x		x
Social support	The 20-item Medical Outcomes Study Social Support Survey Instrument uses multi-trait scaling analyses of 4 functional support scales and provides an overall functional social support index (Cronbach alpha=0.91) [45].	x	x		x		x
Medication self-efficacy	The 13-item Self-Efficacy for Appropriate Medication Use Scale measures how confident individuals are in taking their medications correctly in certain situations (Cronbach alpha=0.89) [46].	x	x		x		x
Depression	The 9-item Patient Health Questionnaire is used to diagnose and measure the severity of depression as well as monitor response to treatment (Cronbach alpha=0.89) [47].	x	x		x		x
Anxiety	The 7-item Generalized Anxiety Disorder Assessment is used to screen and measure severity of generalized anxiety disorder (Cronbach alpha=0.92) [48].	x	x		x		x
Post-traumatic stress disorder	The 17-item Post-Traumatic Stress Disorder Checklist: Civilian is a popular self-report rating scale for post-traumatic stress disorder. The civilian version will be used instead of the military version because the majority of participants will not have been on recent active duty, >35% of Veterans with post-traumatic stress disorder had a nonmilitary trauma, and non-Veteran women will be included (29%; Cronbach alpha=0.87) [49].	x	x		x		x

#### Process Evaluation

Upon completion of the intervention study, we will conduct a process evaluation so that improvements can be made for a multi-site full scale study. The objectives of the process evaluation are to assess participants’ perspectives on feasibility, acceptability, usability, challenges, strengths, and weaknesses of the intervention. In addition, we will ascertain reasons for nonadherence to antiplatelet medications since understanding the reasons for nonadherence is crucial to avoid negative patient outcomes. We will adopt a qualitative research approach using in-depth individual interviews from a diverse sample of participants (total n=20).

#### Analysis

Descriptive statistics, means, and SDs for quantitative variables, and frequencies and percentages for categorical variables, will be provided for all demographic and study variables. Random assignment of the patients to the text messaging, mobile app, and website groups should ensure that there are no preexisting differences between the three groups in personal factors (eg, sociodemographic, clinical, and psychosocial characteristics).

#### Medication Adherence

The primary outcome will be the percentage of prescribed doses taken based on MEMS data (aggregated over 12 months). Secondary outcomes will include: (1) percentages of days correct number of doses were taken, (2) doses taken on schedule, (3) MMAS-8, (4) 7-day recall, (5) VA Corporate Data Warehouse (Veterans), and (6) Peoplechart Meds Incontext (non-Veterans). The MMAS-8 will be analyzed as a range of 0-8. We will synthesize adherence measures with the key primary endpoint of MEMS data compared with secondary measures of adherence to assess for concurrent and predictive validity. We will also calculate associations between all of the adherence measures but expect that they will track well together. Prior to fitting the mixed-effects linear model, we will examine univariate and bivariate relationships between the outcomes and both demographic and coded qualitative responses.

#### Primary Statistical Analysis

Our primary analysis will use a mixed-effects linear model. The predictors will be a categorical predictor of group (with control as reference), time, and the group-by-time interactions, as well as the recruitment site (SFVAMC vs JMMC), as randomization is stratified by site. The parameters in the group-by-time interaction are the primary parameters of interest, describing whether adherence changes differentially over baseline in each of the treatment groups compared to the control group. We will also investigate whether other models for change over time are more appropriate, including arbitrary means or nonlinear time functions through comparison of Akaike information criterion/Bayesian information criterion. Secondary analyses will adjust for select covariates that are strongly associated with adherence.

#### Predictors of Medication Adherence

To determine the factors related to medication adherence, we will examine sociodemographic, clinical, and psychosocial variables. The variables that show at least a moderate correlation and have a significant relationship to medication adherence (*P*<0.10) will be included in a multiple regression analysis in which medication adherence is the dependent variable. The multiple regression analysis will provide the optimum combination of these variables to explain the total percent of variance in medication adherence, and indicate the unique contribution of each variable to the regression model.

#### Process Evaluation

The in-depth individual interviews for process evaluation will be transcribed, coded, and analyzed in the same manner as in Aims 1 and 2. We will explore reasons for attrition over the 12-month period. We will complete field notes after the conclusion of each interview, and all interviews will be transcribed verbatim. Thematic analysis will include reading the completed field notes and interview transcripts. The key outcomes will be related to patient satisfaction and process evaluation, particularly focusing on how views vary across groups.

## Results

We began enrollment for the Mobile4Meds focus groups in January 2017. The focus group data will inform the development of the RCT, which is scheduled to begin in early 2018. We anticipate this four-year research study will be completed in late 2020.

We believe that participants will provide valuable contributions on the content and frequency of text messaging in Aim 1. We expect that focus groups that are stratified by low/high mobile phone use and sex will have differing opinions on frequency of text messaging delivery; however, we also predict that the perceived usefulness of text messaging content will be similar across groups. Based on participants’ responses, we will refine our text messaging content and frequency in preparation for Aim 3.

By conducting Aim 2, we will complete a content analysis of the top 10 commercially available mobile apps that will provide an evidence-based guide for health researchers and clinicians. We expect that using the top 2-4 selected mobile apps over 2-4 weeks on participants’ personal smartphones will provide clear insight into which mobile app should be used for the RCT.

This study is powered to prove the efficacy of using different mobile strategies to promote adherence to antiplatelet medications. By conducting Aim 3, we will compare two mHealth strategies (with a website-control condition) among Veterans using multiple methods to assess adherence. We will also examine the relationship between medication adherence and important variables (ie, health-related quality of life, social support, self-efficacy, depression, anxiety, and post-traumatic stress disorder). The study duration of 12 months, which is the recommended duration of antiplatelet use for most MI/PCI patients, will provide valuable information about patterns of medication adherence.

## Discussion

Evaluating the clinical application of mHealth strategies through rigorous scientific design and methodology is an important step in establishing whether there is potential for long-term benefit among Veterans who live with chronic disease [50]. The best way to ensure strict medication adherence in patients has continued to elude health care providers. Worldwide development and adoption of mobile technology has successfully spurred dramatic growth of mHealth as a platform to deliver intervention strategies for various health conditions in the past several years [51].

Researchers suggest that patient knowledge, self-monitoring, counseling, accountability, and a personalized program can contribute to improvement in medication adherence [52]. Experts and multiple stakeholders in medication adherence agree that a patient-centered approach is necessary, and increasing patient knowledge is critically important [25]. Technological solutions have been suggested to alleviate medication nonadherence, such as mHealth technology, electronic monitors, pill-monitoring technology, online resources, and social media [52]. The best manner to engage with Veterans via text messaging and mobile apps has yet to be determined among those of different ages, sexes, chronic diseases, mobile phone usage patterns, and ethnic populations. There are many opportunities to explore these unanswered questions in future studies.

### Limitations

There are potential limitations associated with this study. In Aims 1 and 2, if we do not reach saturation of ideas or if we find distinct differences between groups, we may add a mixed-group to tease out differences. We may also add an additional focus group of mixed and unstratified participants to identify differences. Alternatively, we will conduct structured one-on-one interviews to acquire richer information as a supplement to the focus groups.

In Aim 3, a limitation may be attrition due to Veteran disinterest in receiving text messages or using a mobile app for 12 months. We will address the potential barrier of disinterest during the two focus groups, as planned in Aims 1 and 2, and ask Veterans how to best engage with them after ACS/PCI over 12 months. Multiple strategies will be used to maximize participant retention and decrease missing data, since the intervention is intended to last 12 months. The dropout or missing data rates for the three groups will be examined to see that they are significantly different. Sensitivity analyses will include a complete-case analysis.

Another potential limitation may be cross-over effects for the text messaging and website (control) groups if they use a mobile app concurrently during the RCT. We will request that patients not use any other electronic medication reminders, including on their mobile phones (eg, alarm, app). At the conclusion of the study, we will ask participants in the text messaging and control groups if they used any other electronic reminders. If they have, we will conduct sensitivity analyses as described.

### Conclusions

As the aims of Mobile4Meds are achieved, scientific knowledge and clinical implementation of behavioral change interventions related to mobile technology and medication adherence will be advanced. Improving medication adherence through mHealth technology may have significant effects on lowering morbidity and mortality among Veterans with coronary heart disease. Since poor adherence to long-term therapies severely compromises the effectiveness of treatment, providing innovative solutions through mHealth may provide relief for this critical issue in population health from the perspective of clinical outcomes and quality of life [53]. Moreover, since medication nonadherence is a major global problem [1], using mHealth strategies may be a powerful solution to reach large, diverse populations in a cost-effective manner. In the future, implementing a strategy to promote adherence through the vulnerable period of 12 months post-ACS/PCI may be highly acceptable and generalizable. The findings from Mobile4Meds may also be highly applicable to other patient classes who require long-term treatment and strict medication adherence.

## Appendix

Multimedia Appendix 1 This is the peer-review summary statement for the grant proposal association with this manuscript. The recommended changes were applied to the final grant proposal that was funded.

## Abbreviations

ACS: acute coronary syndrome
JMMC: John Muir Medical Center
MEMS: Medication Event Monitoring System
mHealth: mobile health
MI: myocardial infarction
MMAS-8: Morisky Medication Adherence Scale
PCI: percutaneous coronary intervention
RA: research assistant
RCT: randomized controlled trial
SD: standard deviation
SFVAMC: San Francisco Veterans Affairs Medical Center
