# Supplementary material for: Comparing Mobile Health Strategies to Improve Medication Adherence for Veterans With Coronary Heart Disease (Mobile4Meds): Protocol for a Mixed-Methods Study
Source: JMIR Res Protoc. 2017 Jul 18;6(7):e134. doi: 10.2196/resprot.7327 (PMC5539386; doi:10.2196/resprot.7327)
Supplement: Multimedia Appendix 1 [file resprot_v6i7e134_app1.pdf]

SUMMARY STATEMENT

PROGRAM CONTACT: **(Privileged Communication)** ***Release Date:*** **10/01/2015**

Application Number: **1 IK3 HX002055-01**

Principal Investigator

PARK, LINDA GRACE

Applicant Organization: VETERANS AFFAIRS MED CTR SAN FRANCISCO

Review Group: **NRI1**
NRI Nursing Research Initiative (ORD program, managed by HSR&D)
NRI1 - Nursing Research Initiative

Meeting Date: **08/27/2015** ***RFA/PA:*** **HX15-025**
Council: **OCT 2015**
Requested Start: **01/01/2016**

Project Title: **Mobile Health Strategies for Veterans with Coronary Heart Disease**

SRG Action: **Impact Score: 207**

Human Subjects: **30-Human subjects involved - Certified, no SRG concerns**
Animal Subjects: **10-No live vertebrate animals involved for competing appl.**
Gender: **1A-Both genders, scientifically acceptable**
Minority: **1A-Minorities and non-minorities, scientifically acceptable**
Children: **1A-Both Children and Adults, scientifically acceptable**
Clinical Research - not NIH-defined Phase III Trial

Project Year	Direct Costs Requested
1	203,586
2	271,217
3	271,986
4	251,996
TOTAL	998,785

ADMINISTRATIVE BUDGET NOTE: The budget shown is the requested budget and has not been adjusted to reflect any recommendations made by reviewers. If an award is planned, the costs will be calculated by VA Office of Research and Development (ORD) staff based on the recommendations outlined in the BUDGET COMMENT section and any relevant ORD service-specific limitations.

NOTE TO APPLICANT: No funding decision has been made on this proposal at this time. If a decision is made to fund this proposal, you will be notified to complete the Just-in-Time process at your local VA Medical Center as soon as possible so that there will be no delay in the release of funds. As a reminder, funding is always dependent upon the availability of funds.

KEY SUMMARY POINTS:

1. Innovative study that addresses an important, timely topic in VA with a vulnerable population; Strong investigative and mentorship team; Strong operational support
2. Study leverages the use of cost-effective mHealth strategies; Focus group data is a strength
3. Measurement of outcomes is not clearly defined (see reviewers 1 & 2), and consideration of the potential for cross-over in RCT with publicly available apps is not addressed (see reviewer 2); The statistical plan for Aim 3 requires additional detail including comparisons of groups/conditions, management of missing data, and adjustments for multiple testing
4. Further justification is needed for selection of the two apps in Aim 2; The application does not address app adaptability issues (see reviewer 3)

DESCRIPTION (provided by applicant):
The proposed research seeks to determine whether text messages/messaging (TM) or a mobile application (app), compared with an educational website-control provided to all Veterans, can improve adherence to antiplatelet therapy among patients following acute coronary syndrome or percutaneous coronary intervention (ACS/PCI). Adherence to antiplatelet medications is critical to prevent life-threatening complications (i.e., stent thrombosis); yet rates of non-adherence range from 21-57% by 12 months. Mobile technology through TM or mobile apps is a practical and inexpensive strategy to promote behavior change and enhance medication adherence. The three specific aims of this proposal are to: 1) determine preferences for content and frequency of TM to promote medication adherence through focus groups; 2) determine the most patient-centered app to promote adherence through a content analysis of all commercially available apps for medication adherence and focus groups centered on usability; and 3) compare adherence to antiplatelet medications in 225 Veterans post ACS/PCI through a randomized clinical trial. Participants will be randomized to either TM, mobile app, or website-control group. The focus groups will be stratified by low/high mobile phone use and sex. In addition, the proposed work will provide Dr. Park with the mentorship and experience necessary to establish herself as an early career investigator in the fields of cardiovascular disease prevention and mobile health research. Dr. Park's primary career goal is to become an independent nurse investigator who applies novel technology for patient-centered behavioral interventions to improve clinical outcomes and quality of life for cardiac patients and Veterans. Dr. Park has assembled an outstanding interdisciplinary team of mentors with expertise in the required areas of training. Eileen Collins, PhD, RN will serve as Primary Nurse Mentor; Mary Whooley, MD will serve as VA On-Site Mentor; and Janet Shim, PhD will serve as Qualitative Research Mentor. In addition, John Boscardin, PhD will conduct all quantitative statistical analyses related to the study. Consultants include Yoshimi Fukuoka, RN, PhD (expert in mobile health strategies) and Dr. Michael Ho (expert in medication adherence and cardiovascular disease). Susan Woods, MD, MPH (Director of Patient Experience, Connected Health, Office of Information and Analytics, VHA) will serve as a collaborator, particularly in relationship to our use of “Annie,” the new VA automated TM program with a goal to help patients take a more active role in their health. The Nursing Research Initiative will provide Dr. Park with an exceptional opportunity to receive the mentorship and experience necessary to prepare a strong scientific foundation for a compelling Investigator Initiated Research proposal to evaluate clinical outcomes (i.e., cardiac morbidity and mortality) through a multi-site full scale RCT. Determining the efficacy of mobile technology through a Veteran-designed protocol to promote medication adherence will have a significant impact on Veteran health and public health, particularly for individuals with chronic diseases that require strict medication adherence.

CRITIQUE 1

1. Significance (including Importance of the Problem Addressed).

Improvement of medication adherence is important to the health of Veterans with coronary heart disease. Understanding the use of mobile health technologies and effect on health of veterans with chronic diseases is critical. The use of this technology may provide and innovative, practical and inexpensive approach to improve medication adherence.

2. Approach (including Feasibility).

This is a very well written and ambitious proposal. This is a mixed-method study using both qualitative and quantitative methods.
The approach for recruitment and analysis for aims 1 and 2 is well described. The identification of the top 2 apps for medication adherence needs to be justified, why 2? It isn't clear how analysis would lead to the clear cut identification of just 2 apps.

For AIM 3, the analysis section needs to be clarified and more detailed for all the outcomes that will be assessed. If there is an overall group differences what are the comparisons of interest between the 3 arms, and what adjustments for multiple comparisons will be done. Is the assumption that all outcomes are normally distributed? Adherence outcomes can be skewed as well as utilization outcomes.

There are many adherence measures proposed, how will results of analysis for all of these be synthesized? What sensitivity analysis is proposed?

The sample size calculation needs more detail on assumptions and what methods were used for calculating power, t-test, ANOVA? Are there adjustments for multiple testing? Does the proposed effect size detect a difference between each treatment and the attention control or what comparison does it represent? Some mapping to the adherence measure would be helpful to assess clinical implications.

The use of a linear mixed model (LMM) has a less stringent assumption on missing data than completely missing at random, general linear mixed models via maximum likelihood estimation, and implicitly accommodates missing-ness when missing-ness is due either to treatment, to prior outcome, or to other baseline covariates included in the model, defined as missing at random.

3. Impact and Innovation.

The design of the study is innovative and results of the study have the potential to have an impact on the health of veterans with coronary heart disease.

4. Investigators and Environment (including Investigator Qualifications, and Facilities and Resources).

This is an accomplished and qualified investigator team. The resources and environment are excellent for this study.

5. Mentor Qualifications and **Mentoring Plan**.

This is an excellent and well qualified mentoring team.

6. Response to Comments from Previous Scientific Review.

N/A

7. Human Subject Protections.

No concerns

8. Inclusion of Women and Minorities.

No concerns. Oversampling to include more women in the study.

9. Care and Use of Vertebrate Animals in Research.

N/A

10. Budget.

No concerns.

11. Overall Impression.

This is a potentially important study that could provide important information to the VA and have a significant impact on veteran health. The analysis section needs more detail and clarification to be able to determine feasibility. The synthesizing of the results of all the adherence outcomes needs to be detailed.

12. Key Strengths.

1. Innovative, ambitious study on important topic.

2. Strong research team

3. Use of mHealth strategies

13. Key Weaknesses.

1. Analysis section needs clarification and detail

2. Justification for selecting just 2 apps for AIM 2

3. Missing data process needs more detail and justification

CRITIQUE 2

1. Significance (including Importance of the Problem Addressed).

Acute coronary syndrome events and percutaneous coronary interventions are common among VA patients. Adherence to medications that are guideline recommended after these scenarios is suboptimal. Interventions that improve adherence are likely to reduce morbidity and mortality from these diseases. Mobile technology is a promising low-cost, convenient modality for delivering interventions that might improve adherence to medications during the critical 12 months after these coronary heart disease scenarios.

2. Approach (including Feasibility).

This is a mixed methods series of studies recruiting patients recently status post ACS/PCI for the following investigations: 1) 4 focus groups of 4-6 patients to ascertain optimal text messaging content and frequency for improving post ACS/PCI medication adherence, 2) investigator evaluation of existing apps for medication adherence followed by 4 focus groups of 4-6 patients to provide feedback on the top 2 apps followed by 1 week crossover testing of the 2 apps followed by repeat focus groups to obtain further feedback and choose the favorite app, and 3) a 3-arm RCT testing the TM vs the preferred app vs control web-site intervention.

The proposal describes very applicable preliminary data to support the research but no quantitative results regarding the efficacy of TM or apps for medication adherence are given—statistical significance is given but the reader cannot determine if results are clinically meaningful. Review of the corresponding published articles shows that adherence was increased by about 15% with antiplatelet medications in the PI's preliminary study, but not statistically significantly with statin medications so the PI is appropriately focusing the current proposal on antiplatelet medications. There is very good attention to VA policies, trends and issues throughout the text.

The patient population is very targeted and appropriate for investigation considering the prevalence and circumstances of medication taking post ACS/PCI. The proposed methods for recruitment are mostly adequate. To enhance recruitment of women, the PI proposes to recruit from a non-VA facility where she has worked. This is important to enhance generalizability to women but the ability to use VA funds for this purpose needs to be confirmed. In addition, the feasibility needs to be confirmed because there are conflicting accounts in the text as to the ACS/PCI patient pool (at one point 110 patients per year is mentioned, which seems a small pool if trying to recruit only women from it). It would also be helpful to know how well the women from the non-VA facility compare demographically to VA women patients.

The methods for the focus groups seem appropriate and likely to elicit meaningful results. It seems like slightly larger groups, e.g., 6-8 participants, might allow a better distribution of age, race and SES and thus be more generalizable. Alternatively, structured one-on-one interviews with a corresponding total N of participants might provide richer information. The utility of focus groups for text messaging is a bit unclear if the VA is already proceeding with 'Annie', which is proposed to be used in the RCT. It may be that Annie is simply the conduit for text messages? This should be clarified. The timelines for conducting and completing transcription and analyses for the focus groups (1 year) seems ambitious.

The methods for the RCT are also solid with a few exceptions. There seems ample opportunity for crossover of the TM and control groups into the mobile app group given that the apps will be commercially available. The consent process may need to be sufficiently vague to avoid this. Given that medication adherence can be difficult to assess accurately, the proposal has smartly planned to use several redundant methods, including 2 objective methods, MEMS Caps and pharmacy records. However, the primary outcome for the RCT is not specified and the specific outcome for several of the measurements (e.g., Moriskey scale, MEMS caps, pharmacy record data) is not specified either. This is particularly important for the sample size calculation. One of the objective measures, probably the MEMS caps given the duration of the study, would be the preferable primary outcome.

I am surprised by the use of the civilian vs the military version of the PCL-C given that PTSD in veterans will stem very predominantly from military trauma.

The statistical analyses appear generally appropriate albeit sparsely described. The assumption that missing data will be MCAR is a bit of a leap. Missing data in behavioral intervention trials is more likely MAR and can be addressed by adjusting for factors related to the missing-ness. The use of survival analysis seems ambitious given the study is not close to be powered adequately for clinical outcomes; however, it may be useful simply for didactic purposes.

A couple of overarching concerns about the proposed research are 1) that the results might be obsolete as soon as they are disseminated given that new apps are developed frequently and 2) whether the VA can endorse or subsidize use of any one app should that prove the most successful in the RCT. Text addressing these limitations should be added.

3. Impact and Innovation.

A better understanding of the acceptability and preference of TM and apps for medication adherence in Veterans, as will be gleaned from the focus groups, would be welcome information. If shown to be more effective for adherence than the control intervention in the RCT, this information alone could be valuable. Even more valuable would be the information from the potential multi-site RCT with clinical outcomes. The use of the technology is innovative in the VA and potentially very efficient, inexpensive and convenient, if effective.

4. Investigators and Environment (including Investigator Qualifications, and Facilities and Resources).

The PI's background and experience are very appropriate for this study. Dr Park has performed a systematic review of studies of mobile technology for medication adherence and a pilot RCT of text messaging for medication adherence post ACS/PCI. She appears to have assembled the necessary mentors, collaborators, and resources to complete the proposed research.

5. Mentor Qualifications and **Mentoring Plan**.

The mentors have the adequate background expertise and experience to help guide the PI on this project. The application includes a detailed mentoring plan with adequate meeting frequency with mentors who have appropriate expertise to advise the PI.

6. Response to Comments from Previous Scientific Review.

Not applicable.

7. Human Subject Protections.

Adequate.

8. Inclusion of Women and Minorities.

Very adequate.

9. Care and Use of Vertebrate Animals in Research.

Not applicable.

10. Budget.

The budget seems appropriate except there does not appear to be budget for smart phones for those patients who do not have them. Also the project manager is listed as 10% effort in the table but 20% in the text.

11. Overall Impression.

This is a really well-written and thought out proposal with a logical sequence of studies that should address an important problem in a meaningful way. One major issue is that the results might be dated as soon as they are disseminated given that new mobile phone apps are developed constantly. There are a few clarifications or enhancements that could be made regarding defining the main outcomes and statistical analysis.

12. Key Strengths.

1. Important area to address in middle aged and older Veterans.

2. Well-sequenced series of projects to build an intervention to improve medication adherence.

3. Intervention has potential for effectiveness plus low cost and high convenience.

4. The PI has solid background to lead the projects and has assembled appropriate mentors and collaborators

13. Key Weaknesses.

1. If the mobile app proves to be the most effective of the 3 interventions, it could become out of date quickly. The RCT might require repeating with new or improved apps.

2. The primary outcome is not specified and none of the outcomes are defined clearly.

3. Statistical analysis could be better detailed.

4. Cross-over is a potential issue in the RCT.

5. Click here to enter text.

CRITIQUE 3

1. Significance (including Importance of the Problem Addressed).

Significance of the issue (medication adherence post Cardiac event) has been well established and is a critical need to be addressed.

2. Approach (including Feasibility).

The PI has thoroughly explicated each step of the proposal and has included back up plans for recruitment. Each component of the collection, analysis and dissemination is supported.

3. Impact and Innovation.

The findings for each specific aim have the potential to recraft quality, cost-efficient and effective care delivery. The portion regarding the building of a repository of effective/preferred TM has enormous potential within and beyond the VA care delivery environment and is especially salient in the ACA environment.

4. Investigators and Environment (including Investigator Qualifications, and Facilities and Resources).

The facilities to be used by the PI along with her research and publication record support her ability to complete this ambitious study.

5. Mentor Qualifications and **Mentoring Plan**.

Exceptional qualified and broad group of mentors addressing every aspect of the proposal.

6. Response to Comments from Previous Scientific Review.

NA

7. Human Subject Protections.

Noted with the proposal.

8. Inclusion of Women and Minorities.

Specifically noted within the recruitment section.

9. Care and Use of Vertebrate Animals in Research.

NA

10. Budget.

Specific contracts for smart phones and data plans are not readily visible within the budget.

11. Overall Impression.

Outstanding proposal that has the potential to change and improve practice and health outcomes for a fragile population.

12. Key Strengths.

1. Research plan well thought through.

2. Data collection plan with back up strategies for participant enrollment.

3. Mentorship plan and the track record of working with an exceptional group of mentors.

13. Key Weaknesses.

1. Expensive plan with the majority of costs being personnel.

2. Very little justification of using TM, apps with a population who may have visual impairment and who may have difficulty using a smart phone/touch screen.

3. Given the direct applicability, a dissemination plan that focuses so heavily on journal publication misses an opportunity. Presentation beyond the facility – at the VISN level and with a focus on providers may lead to more expedient implementation.

MEETING ROSTER

NRI Nursing Research Initiative (ORD program, managed by HSR&D)
Health Services Research Parent IRG
Office of Research & Development
NRI1 - Nursing Research Initiative
NRI1 R
August 27, 2015

CHAIRPERSON

ERSEK, MARY T., PHD
DIRECTOR, NATIONAL PROMISE CENTER
PHILADELPHIA VA MEDICAL CENTER
PROFESSOR - CLINICIAN EDUCATOR
UNIVERSITY OF PENNSYLVANIA SCHOOL OF NURSING
PHILADELPHIA, PA 19104

MEMBERS

COFFMAN, CYNTHIA J PHD *
BIOSTATISTICIAN
DURHAM VA HSRD CENTER OF INNOVATION
ASSISTANT RESEARCH PROFESSOR
DEPARTMENT OF BIOSTATISTICS AND BIOINFORMATICS
DUKE UNIVERSITY MEDICAL CENTER
DURHAM, NC 27705

DONLEY, ROSEMARY , BSN, PHD *
PROFESSOR
JACQUES LAVAL ENDOWED CHAIR FOR JUSTICE
FOR VULNERABLE POPULATIONS
DUQUESNE UNIVERSITY SCHOOL OF NURSING
PITTSBURGH, PA 15282

HIGGINS, PATRICIA A., RN, BSN, PHD
ASSOCIATE PROFESSOR
DEPARTMENT OF NURSING
FRANCES PAYNE BOLTON SCHOOL OF NURSING
CASE WESTERN RESERVE UNIVERSITY
CLEVELAND, OH 44106

LING, CATHERINE G. *
ASSISTANT PROFESSOR
PHD AND FNP/DNP PROGRAMS
DANIEL K. INOUYE GRADUATE SCHOOL OF NURSING
UNIFORMED SERVICES UNIVERSITY
OF THE HEALTH SCIENCES
BETHESDA, MD 20814

PATRICIAN, PATRICIA A. PHD *
DONNA BROWN BANTON ENDOWED PROFESSOR
SCHOOL OF NURSING
UNIVERSITY OF ALABAMA AT BIRMINGHAMV
BIRMINGHAM, AL 35487

SAMAR, ANNMARIE D, PHD *
PROFESSOR
DEPARTMENT OF NURSING
FRAMINGHAM STATE UNIVERSITY
FRAMINGHAM, MA 01702

UPHOLD, CONSTANCE R. RN, PHD
ASSOC DIRECTOR, IMPLEMENTATION & OUTCOMES RES
GERIATRIC RESEARCH EDUCATION CLINICAL CENTER
GAINESVILLE VA MEDICAL CENTER
ASSOCIATE PROFESSOR OF AGING & GERIATRICS
UNIVERSITY OF FLORIDA COLLEGE OF MEDICINE
GAINESVILLE, FL 32608

WU, WEN-CHIH , MD
STAFF CARDIOLOGIST
PROVIDENCE VA MEDICAL CENTER
ASSOCIATE PROFESSOR
DEPARTMENT OF MEDICINE
BROWN UNIVERSITY MEDICAL SCHOOL
PROVIDENCE, RI 02908

YANCY, WILLIAM S. JR., MD *
RESEARCH SCIENTIST
DURHAM VA CENTER OF INNOVATION
DIRECTOR, DUKE UNIVERSITY DIET AND FITNESS
CENTER
ASSOCIATE PROFESSOR OF MEDICINE
DUKE UNIVERSITY MEDICAL CENTER
DURHAM, NC 27705

SCIENTIFIC REVIEW OFFICER

KARRAS, ELIZABETH PHD
DEPARTMENT OF VETERANS AFFAIRS
VETERANS HEALTH ADMINISTRATION
OFFICE OF RESEARCH AND DEVELOPMENT
HEALTH SERVICES RESEARCH AND DEVELOPMENT
WASHINGTON, DC 20420

KELLEY, PATRICIA WATTS, RN, PHD
DEPARTMENT OF VETERANS AFFAIRS
VETERANS HEALTH ADMINISTRATION
OFFICE OF RESEARCH AND DEVELOPMENT
HEALTH SERVICES RESEARCH & DEVELOPMENT
WASHINGTON, DC 20420

* Temporary Member. For grant applications, temporary members may participate in the entire meeting or may review only selected applications as needed.

Consultants are required to absent themselves from the room during the review of any application if their presence would constitute or appear to constitute a conflict of interest.
